# Identification of super-enhancer-associated transcription factors regulating glucose metabolism in poorly differentiated thyroid carcinoma

**DOI:** 10.1590/1678-4685-GMB-2021-0370

**Published:** 2022-09-19

**Authors:** Kun Liu, Yongrui Du, Hui Li, Xuexia Lin

**Affiliations:** 1 Tianjin Hospital, Endocrinology Department, Tianjin, P. R. China.; 2 80th Group Military Hospital, Chinese Peoples Liberation Army, Endocrinology Department, Weifang, Shandong, P. R. China; 3 XingTai Medical College, Basic Experiment Center, Xingtai, Hebei, P. R. China

**Keywords:** Poorly differentiated thyroid carcinoma, glucose metabolism, super-enhancer, transcription factor, prognosis

## Abstract

This study aimed to uncover transcription factors that regulate super-enhancers involved in glucose metabolism reprogramming in poorly differentiated thyroid carcinoma (PDTC). TCA cycle and pyruvate metabolism were significantly enriched in PDTC. Differentially expressed genes in PDTC vs. normal control tissues were located in key steps in TCA cycle and pyruvate metabolism. A total of 23 upregulated genes localized in TCA cycle and pyruvate metabolism were identified as super-enhancer-controlled genes. Transcription factor analysis of these 23 super-enhancer-controlled genes related to glucose metabolism was performed, and 20 transcription factors were obtained, of which KLF12, ZNF281 and RELA had a significant prognostic impact. Regulatory network of KLF12, ZNF281 and RELA controlled the expression of these four prognostic target genes (LDHA, ACLY, ME2 and IDH2). *In vitro* validation showed that silencing of KLF12, ZNF281 and RELA suppressed proliferation, glucose uptake, lactate production and ATP level, but increased ADP/ATP ratio in PDTC cells. In conclusion, KLF12, ZNF281 and RELA were identified as the key transcription factors that regulate super-enhancer-controlled genes related to glucose metabolism in PDTC. Our findings contribute to a deeper understanding of the regulatory mechanisms associated with glucose metabolism in PDTC, and advance the theoretical development of PDTC-targeted therapies.

## Introduction

Thyroid carcinoma (TC) is a common endocrine-related tumor with an increasing incidence (Bray *et al.*, 2018; Wu *et al.*, 2018). TC can be divided into differentiated thyroid carcinoma (DTC), poorly differentiated thyroid carcinoma (PDTC) and anaplastic thyroid carcinoma (ATC) (Nucera *et al.*, 2009). DTC can be further divided into follicular thyroid carcinoma (FTC), papillary thyroid carcinoma (PTC) and follicular variant of papillary thyroid carcinoma (FVPTC) subtypes (Nucera *et al.*, 2009; Acquaviva *et al.*, 2018). PDTC accounts for 2%-15% of all TC, with strong invasiveness, heterogeneity and high local recurrence rate (Sanders *et al.*, 2007; Jemal *et al.*, 2011; Ibrahimpasic *et al.*, 2019). To date, there is no standard treatment option for PDTC. Surgery is the preferred management option for PDTC. Effectiveness of adjuvant treatments such as radiotherapy and chemotherapy for PDTC is unclear (Ibrahimpasic *et al.*, 2019). Specific pathogenesis of PDTC is still in the exploratory stage. Exploring the mechanisms regulating the progression of PDTC is necessary to advance the prevention and treatment strategies.

Evidence of the important roles of glucose metabolism in cancer development is increasing (Pavlova and Thompson, 2016; Abdel-Wahab *et al.*, 2019; Ghanavat *et al.*, 2021; Rodríguez *et al.*, 2021). Abnormally enhanced glucose metabolism is one of the hallmarks of tumor cells (Hay, 2016). As an important source of carbon and energy for cells, glucose affects cellular function through the production of energy and different glucose derivatives through metabolic pathways such as the glycolysis, TCA cycle, the pentose phosphate pathway and the pyruvate metabolic pathway. Modulation of cellular metabolism is one of the ideas in clinical cancer treatment. The rise of targeted therapy provides the basis for advancing cancer treatment (Elf and Chen, 2014). Targeting the metabolic differences between tumor cells and normal cells has promised application prospect (Park *et al.*, 2020). In recent years, glucose metabolism has been broadly concerned in TC. Glucose metabolism not only affects the growth and invasion of thyroid cancer cells, but also has a complex association with their differentiation (Grabellus *et al.*, 2012; Suh *et al.*,2019; Heydarzadeh *et al.*, 2020). However, the molecular mechanisms of glucose metabolism regulating PDTC are not well defined. In-depth study of glucose metabolism in PDTC is necessary for the discovery of novel therapeutic targets.

Abnormal metabolism of tumor cells is dependent on the overactive transcription of oncogenes (Boroughs and Deberardinis, 2015; Marbaniang and Kma, 2018). Super-enhancers are dense regions of enhancers on the genome, have been widely documented to be important regulatory elements which drive oncogene expression (Sengupta and George, 2017; Tang *et al.*, 2020). Strategies that target super-enhancer-associated regulation, for example, inhibition of super-enhancer-bound transcription factors or other cofactors, have shown promising therapeutic results. Therefore, it is valuable to identify key super-enhancer-controlled genes and their transcription factors.

In the current study, we characterized glucose metabolism-related super-enhancer-controlled genes, and investigated the prognostic value of transcription factors that regulate these genes in PDTC. Furthermore, the significance of these key transcription factors on proliferation and glucose metabolism in PDTC cells was confirmed *in vitro*. The purpose of this study was to explore transcription factors that regulate super-enhancers-controlled genes associated with glucose metabolism in PDTC.

## Material and Methods

### Research design and data processing

**Figure 1.**
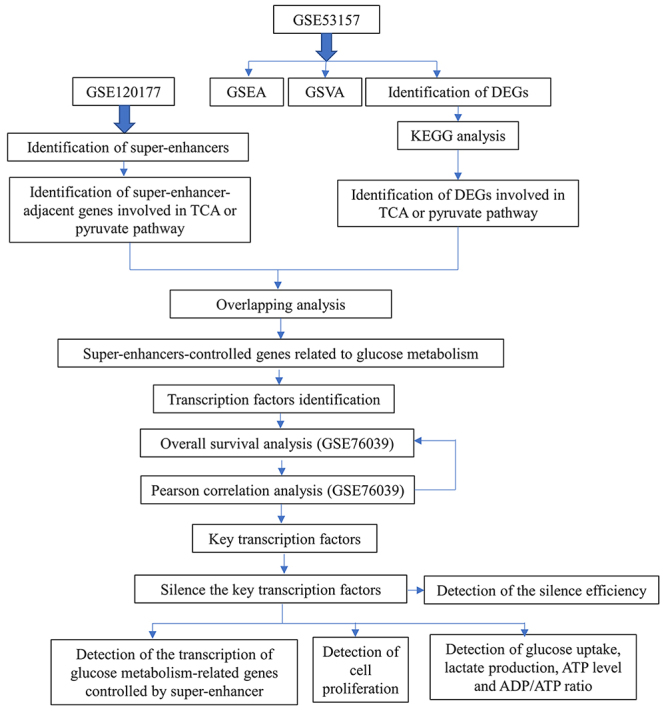
Flowchart of the research design.

The overview of the research design is shown in Figure 1. Datasets for GSE120177, GSE53157 and GSE76039 were downloaded from GEO database. GSE53157 datasets comprised 5 PDTC, 4 FTC, 8 FVPTC, 7 PTC and 3 normal thyroid samples. GSE76039 datasets comprised 32 PDTC or ATC samples and the enrolled patients should have complete clinical information and prognostic data.

### Differentially expressed genes (DEGs) identification

Dataset GSE53157 was used for DEGs identification using the “limma” R package. Genes with log2 fold change absolute value (|Log2 FC|) ≥1.0 and adjusted P< 0.05 were considered as DEGs. DEGs were arranged in descending order of the value of |Log2 expression|.

### Glucose metabolism-related pathways analysis

GSE53157 dataset was used for Gene Set Enrichment Analysis (GSEA). GSEA was applied using GSEA v. 4.0.3 with 13 glucose metabolism-related gene sets retrieved from Molecular Signatures Database (MSigDB, http://www.broad.mit.edu/gsea/msigdb/) (Subramanian *et al.*, 2005). The screening standard was normalized enrichment score≥1, nominal P<0.05 and FDR<0.25.

TCA cycle and pyruvate metabolism scores of 5 PDTC, 4 FTC, 8 FVPTC and 7 PTC in GSE53157 were calculated using the gene set variation analysis (GSVA) algorithm (Hänzelmann *et al.*, 2013). Location of DEGs in TCA cycle and pyruvate metabolism pathway were analyzed using Kyoto Encyclopedia of Genes and Genomes (KEGG) Mapper online tool (https://www.kegg.jp/).

### Super-enhancer and super-enhancer-adjacent gene identification

Histone H3K27 acetylation (H3K27ac) ChIP-seq data of dataset GSE120177 were used for enhancer identification. Enhancers were identified as the regions with abundant of H3K27ac using “findPeaks” parameter of HOMER software. Enhancers were arranged in ascending order of H3K27ac signal using “super enhancer” parameter of HOMER software. Cutoff value was the slope of enhancer curve = 1. Enhancers with a slope greater than 1 were identified as super-enhancers (Tang *et al.*, 2020). Gene closest to a super-enhancer on genome was defined as a super-enhancer-adjacent gene.

### Transcription factor identification

Toolkit for Cistrome Data Browser (http://dbtoolkit.cistrome.org) with default parameters was applied to screen transcription factors of super-enhancer-upregulated genes.

### Overall survival and Pearson correlation analysis

Dataset GSE76039 comprised 32 PDTC or ATC cases was used for overall survival and Pearson correlation analysis. Co-expression networks are visualized using Cytoscape (http://www.cytoscape.org) software. Patients were divided into high and low expression groups based on the median mRNA expression level. Kaplan-Meier curves and log-rank test were performed in this study.

### Cell culture

One normal thyroid epithelial cell line, Nthy-ori 3-1, was obtained from Cell Bank of Chinese Academy of Sciences (Shanghai, China). Two PDTC cell lines, CAL-62 and BHT-101, were obtained from National Infrastructure of Cell Line Resource (Beijing, China). Cells were cultured in Dulbecco’s modified Eagle’s medium (DMEM) (Gibco, NY, USA) with 10% FBS at 37°C.

### Cell transfection

Si-KLF12, si-ZNF281, si-RELA, si-NRF1 and si-negative control (si-NC) were synthesized by Genomeditech (Shanghai, China). These siRNAs were individually transfected into CAL-62 and BHT-101 cells using Lipofectamine 3000 (Invitrogen, MD, USA) for 48 h at 37 °C as the protocol of the manufacturer.

### RNA extraction and qRT-PCR

TRIzol reagent (Invitrogen) was used for total RNA extraction. cDNA was reverse transcribed using First Strand cDNA Synthesis Kit (TaKaRa Bio, Otsu, Japan). qRT-PCR was performed using SYBR Premix Ex Taq II kit (TaKaRa Bio) with GAPDH as the internal control. Relative expression was calculated using 2^−ΔΔCt^ method. The primer sequences were designed as below:

KLF12, 5’-CGGCAGTCAGAGTCAAAACAG-3’ (F) and 5’-GGGAGGATGAAACGGCAGTAG-3’ (R). 

ZNF281, 5’-TAGTGCAGAACCTGGGTCATC-3’ (F) and 5’-ACACGGTAGGCATTTCTACTGA-3’ (R). 

RELA, 5’-ATGTGGAGATCATTGAGCAGC-3’ (F) and 5’-CCTGGTCCTGTGTAGCCATT-3’ (R).

NRF1, 5’-AGGAACACGGAGTGACCCAA-3’ (F) and 5’-TATGCTCGGTGTAAGTAGCCA-3’ (R).

LDHA, 5’-ATGGCAACTCTAAAGGATCAGC-3’ (F) and 5’-CCAACCCCAACAACTGTAATCT-3’ (R).

ACLY, 5’-TCGGCCAAGGCAATTTCAGAG-3’ (F) and 5’-CGAGCATACTTGAACCGATTCT-3’ (R).

ME2, 5’-ATGTTGTCCCGGTTAAGAGTAGT-3’ (F) and 5’-ACCAAGCATTTGTCGTTCTTGT-3’ (R).

IDH2, 5’-CGCCACTATGCCGACAAAAG-3’ (F) and 5’-ACTGCCAGATAATACGGGTCA-3’ (R).

GAPDH, 5’-GGAGCGAGATCCCTCCAAAAT-3’ (F) and 5’-GGCTGTTGTCATACTTCTCATGG-3’ (R).

### Protein extraction and western blotting

Total protein of the indicated cells was extracted using RIPA buffer (GenePharma, China), and the protein concentrations were determined using BCA Protein Assay Kit (Beyotime, China). Equal amounts of protein were separated using 10% SDS-PAGE, and transferred to PVDF membrane (Millipore, USA). The membranes were incubated with primary antibodies at 4 °C overnight, followed by incubated with the horseradish peroxidase (HRP)-conjugated secondary antibodies (ab6721, 1:2000, abcam, Cambridge, UK). Finally, protein bands were detected using ECL chemiluminescence detection kit (Beyotime, China). The primary antibodies were as following: anti-KLF12 (ab129459, 1:2000, abcam); anti-ZNF281 (ab101318, 1:2000, abcam); anti-RELA (#3033, 1:2000, Cell Signaling); anti-NRF1 (ab175932, 1:2000, abcam); anti-β-actin (ab8226, 1:2000, abcam).

### Cell proliferation assay

Cell proliferation analysis was performed using Cell Counting Kit 8 (CCK8) (Beyotime, Jiangsu, China). CAL-62 and BHT-101 cells were transfected with si-NC, si-KLF12, si-ZNF281, si-RELA or si-NRF1 for 48 h. Then, cells were plated into 96 well plates and cultured for 0, 24, 48 and 72 h. 10 μL CCK8 reagent were added into each well at each time point, and then incubated at 37 °C for 2 h. Epoch microplate reader (BioTek Instruments, VT, USA) was used to detect the absorbance at 450 nm (A450).

### Glucose uptake assay

Glucose uptake into CAL-62 and BHT-101 cells was detected using the Glucose Uptake Assay Kit (ab136955, abcam) according to the manufacturer’s instruction. Cells with si-NC, si-KLF12, si-ZNF281, si-RELA or si-NRF1 transfection were seeded into 96 well plate, and cultured in serum free medium for 12 h. Cells were harvested and washed with PBS buffer, followed by Krebs-Ringer-Phosphate-Hepes (KRPH) buffer (with 2% BSA) treatment for 40 min, and then 2-deoxyglucose treatment for 20 min. 2-deoxyglucose-6-phosphate is oxidized to NADPH. Absorbance at 412 nm (A412) was detected using an Epoch microplate reader (BioTek). Glucose present was normalized with total protein. Glucose consumption = (Glucose content of cell-free media) - (Gglucose content of each group). Relative glucose uptake = (Glucose consumption of each group) / (Glucose consumption of si-NC group).

### Lactate production, ATP and ADP levels assay

Lactate production of CAL-62 and BHT-101 cells was measured using the L-lactate Assay kit (ab65331, abcam) as the protocol. ADP levels was determined using ADP assay kit (ab83359, abcam). ATP levels were determined using ATP assay kit (ab83355, abcam). Protein concentration was detected for normalization. A450 was measured using Epoch microplate reader (BioTek).

### Statistical analysis

Data were displayed as mean ± SD. Statistical analysis were carried out using one-way analysis of variance (ANOVA) followed by Tukey’s post hoc test and Student’s *t*-test. P<0.05 was considered to be significant difference.

## Results

### Enrichment analysis of glucose metabolism-related pathways in PDTC

**Figure 2.**
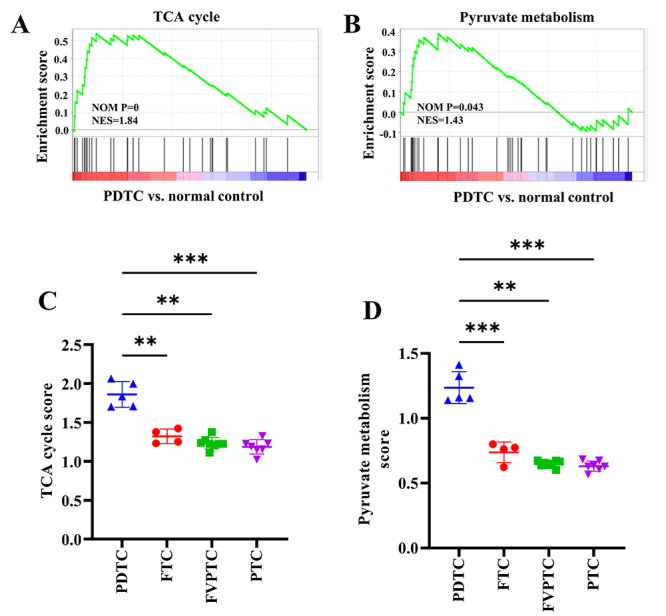
Enrichment of glucose metabolism-related pathway of PDTC. (A/B) GSEA showed that TCA cycle (A) and pyruvate metabolism pathways (B) were significantly enriched. (C/D) TCA cycle (C) and pyruvate metabolism (D) scores of PDTC, FTC, FVPTC and PTC. ANOVA followed by Tukey’s post hoc test was applied to compare the difference among groups. **P<0.01, ***P<0.001.

Five PDTC tissues and three normal control tissues in GSE53157 were analyzed for the enrichment of pathways related to glucose metabolism by GSEA. We found that genes in PDTC were significantly enriched in two pathways including TCA cycle and pyruvate metabolism pathway (Figure 2A and B). Then, 5 PDTC, 4 FTC, 8 FVPTC and 7 PTC samples in GSE53157 cohort were subjected to the TCA cycle score and pyruvate metabolism score. Both TCA cycle score and pyruvate metabolism score of PDTC were significantly higher than those of FTC, FVPTC and PTC (Figure 2C and D). However, there was no notably variation in TCA cycle score or pyruvate metabolism score among FTC, FVPTC and PTC (Figure 2C and D).

**Figure 3.**
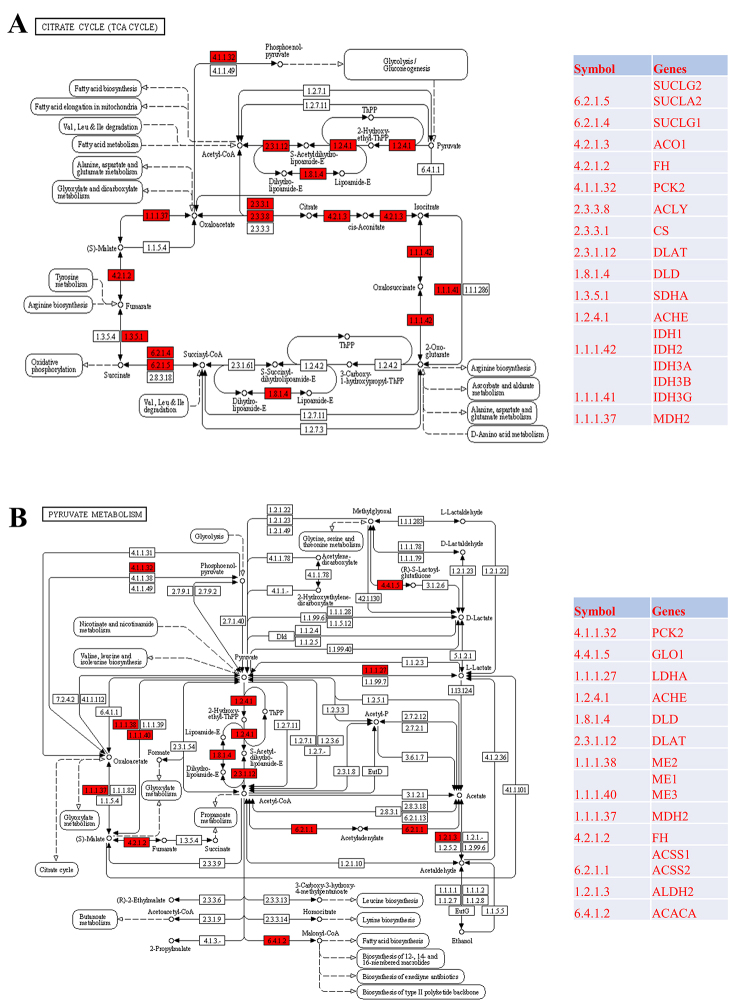
Location of differentially expressed genes in TCA cycle (A) and pyruvate metabolism (B). The red boxes represent the DEGs.

Furthermore, we identified the DEGs in PDTC vs. normal control tissues based on GSE53157, and analyzed the location of DEGs in TCA cycle and pyruvate metabolism. As expected, DEGs were enriched in key reactions in TCA cycle and pyruvate metabolism (Figure 3A and B). PCK2, DLAT, ACHE, DLD, CS, ACLY, MDH2, FH, SDHA, SUCLG1, SUCLG2, SUCLA2, IDH1, IDH2, IDH3A, IDH3B, IDH3G and ACO1 were located in TCA cycle (Figure 3A). PCK2, GLO1, LDHA, ACHE, DLD, DLAT, ME1, ME2, ME3, MDH2, FH, ACSS1, ACSS2, ALDH2 and ACACA were differentially expressed and enriched in the pyruvate metabolism pathway (Figure 3B).

### Super-enhancer-controlled genes related to glucose metabolism in PDTC

**Figure 4.**
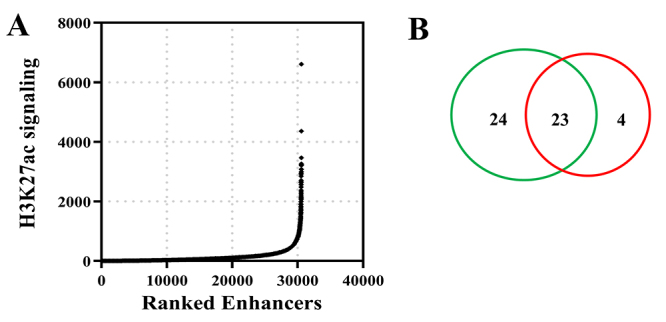
Identification of super-enhancer-controlled genes related to glucose metabolism in PDTC. (A) Super-enhancers were screened in CAL-62 cells using GSE120177 dataset. (B) Venn diagram to show the intersection between super-enhancers-adjacent genes located in TCA cycle or pyruvate metabolism (green circle) and upregulated genes in PDTC located in TCA cycle or pyruvate metabolism (red circle).

Enrichment of H3K27ac is one of the major features of super-enhancers, and is often used in the definition of super-enhancers (Niederriter *et al.*, 2015; Tang *et al.*, 2020). In this study, super-enhancers were identified based on H3K27ac signaling of CAL-62 cells (GSE120177). A total of 537 super-enhancers were identified (Figure 4A). Then, super-enhancer-adjacent genes involved in the TCA cycle or pyruvate metabolism pathway were filtered. As shown in Figure 4B, there were 47 super-enhancer-adjacent genes involved in the TCA cycle or pyruvate metabolism pathway. In addition, we analyzed the differential expression of genes involved in the TCA cycle or pyruvate metabolic pathway in PDTC versus normal controls, and obtained 27 upregulated genes (Figure 4B). Overlapping analysis of the two gene sets described above being performed. The results revealed that 23 super-enhancer-adjacent genes were upregulated in PDTC which located in TCA cycle or pyruvate metabolic pathway (Figure 4B). Therefore, these 23 genes were identified as super-enhancer-controlled genes related to glucose metabolism.

### KLF12, ZNF281 and RELA were identified as the key transcription factors regulating glucose metabolism and PDTC progression

**Figure 5.**
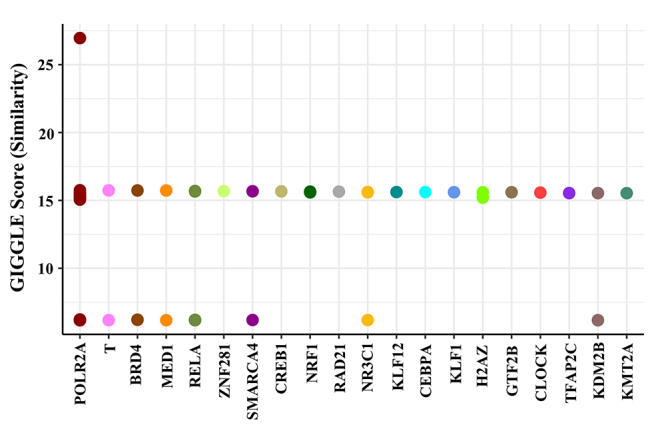
Identification of transcription factors of super-enhancers upregulated genes.

Transcription factors of the 23 super-enhancers-controlled genes were identified. We reasoned that identifying transcription factors that activate super enhancers would allow us to determine whether these *cis* elements are functionally important for PDTC progression. To this end, we evaluated whether specific transcription factor motifs were enriched within super enhancers using the Cistrome Data Browser Toolkit. Twenty transcription factors, including POLR2A, T, BRD4, MED1, RELA, ZNF281, SMARCA4, CREB1, NRF1, RAD21, NR3C1, KLF12, CEBPA, KLF1, H2AZ, GTF2B, CLOCK, TFAP2C, KDM2B and KMT2A, were obtained (Figure 5).

**Figure 6.**
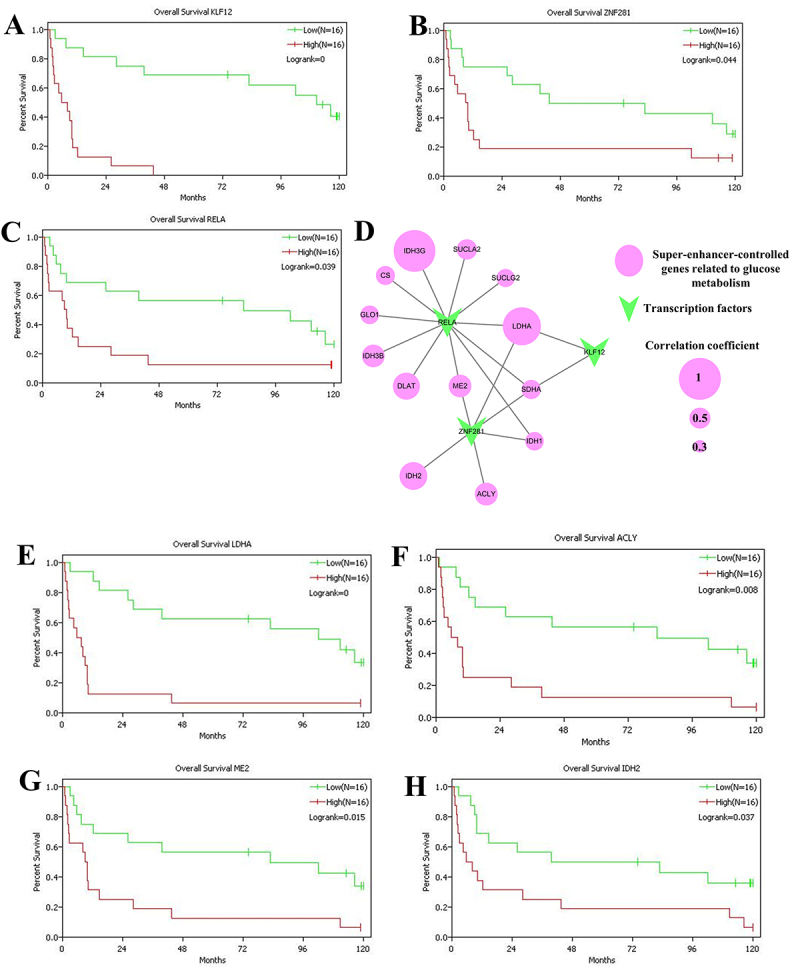
KLF12, ZNF281 and RELA were identified as the key transcription factors in poorly differentiated and anaplastic thyroid carcinoma patients. (A-C) Kaplan-Meier graphs and log-rank test were performed for overall survival analysis of KLF12 (A), ZNF281 (B) and RELA (C) based on GSE76039 cohort. D, Pearson correlation of KLF12, ZNF281 and RELA with super-enhancers-controlled TCA and pyruvate metabolism genes based on GSE76039 datasets. Pink circles represented super-enhancer-controlled genes related to glucose metabolism. Green arrows represented transcription factors. The size of the circles represented the correlation coefficient. (E-H) Kaplan-Meier curves for LDHA (E), ACLY (F), ME2 (G) and IDH2 (H) based on GSE76039 cohort.

Super enhancers are usually driven by a small number of key transcription factors. To further validate the key transcription factors, GSE76039 dataset was derived for overall survival analysis of the 20 transcription factors. High expression of KLF12, ZNF281 and RELA was correlated with an unsatisfactory overall survival in PDTC or ATC patients (Figure 6A-C). Except the above three transcription factors, the other seventeen transcription factors had no significant effect on prognosis of PDTC or ATC patients. Hence, KLF12, ZNF281 and RELA were filtered for further analysis.

To further explore the mechanisms by which the three transcription factors regulate glucose metabolism, we evaluated the Pearson correlation of KLF12, ZNF281 and RELA with super-enhancers-controlled TCA and pyruvate metabolism genes in GSE76039 datasets. As shown in Figure 6D, 13 of the 23 super-enhancer-controlled genes related to glucose metabolism were positively correlated with KLF12, ZNF281 or RELA. Of these, the expression levels of two genes (LDHA and SDHA) were positively correlated with the expression levels of three transcription factors, and two genes (ME2 and IDH1) were positively correlated with the expression of two transcription factors (RELA and ZNF281) (Figure 6D). In addition, we analyzed the relationship of the 13 positively correlated genes and overall survival using GSE76039 dataset. The results showed that high expression of LDHA, ACLY, ME2 and IDH2 was associated with a poor prognosis in PDTC or ATC patient (Figure 6E-H). Hence, LDHA, ACLY, ME2 and IDH2 were selected as the representative glucose metabolism-related genes controlled by super-enhancer. KLF12, ZNF281 and RELA were identified as the key transcription factors.

### Silencing of KLF12, ZNF281 and RELA inhibited proliferation and the expression of super-enhancer-controlled genes in CAL-62 and BHT-101 cells

**Figure 7.**
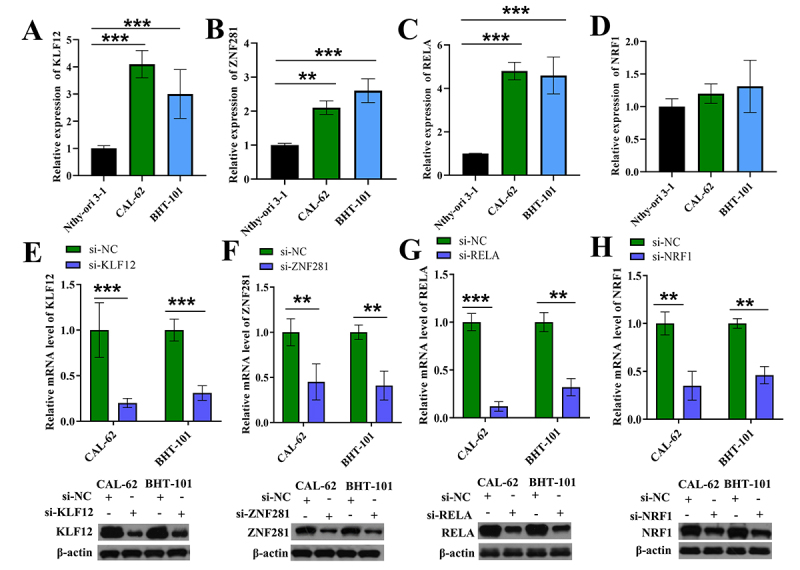
Verification of silencing efficiency of KLF12, ZNF281, RELA and NRF1. (A-D) Expression levels of KLF12 (A), ZNF281 (B), RELA (C) and NRF1 (D) in Nthy-ori 3-1, CAL-62 and BHT-101 cells were measured by qRT-PCR. (E-H) Silencing efficiency of KLF12 (E), ZNF281 (F), RELA (G) or NRF1 (H) in CAL-62 and BHT-101 cells were verified by qRT-PCR and western blotting.

To understand the role of the three prognostic transcription factors, we evaluated the expression levels of KLF12, ZNF281 and RELA in one normal thyroid epithelial cell line and two PDTC cell lines with a non-prognostic transcription factor, NRF1, as a control. We found that the expression of KLF12, ZNF281 and RELA was significantly higher in CAL-62 and BHT-101 cells than Nthy-ori 3-1 cells (Figure 7A-C). However, there was no significant difference of NRF1 expression among CAL-62, BHT-101 and Nthy-ori 3-1 cells (Figure 7D). Subsequently, we established KLF12, ZNF281, RELA and NRF1 silencing cell lines by transfecting si-KLF12, si-ZNF281, si-RELA and si-NRF1 into CAL-62 and BHT-101 cells, respectively. Results of qRT-PCR and western blotting showed that mRNA and protein levels of KLF12, ZNF281, RELA and NRF1 were notably decreased upon si-KLF12, si-ZNF281, si-RELA and si-NRF1 transfection, respectively (Figure 7E-H). 

**Figure 8.**
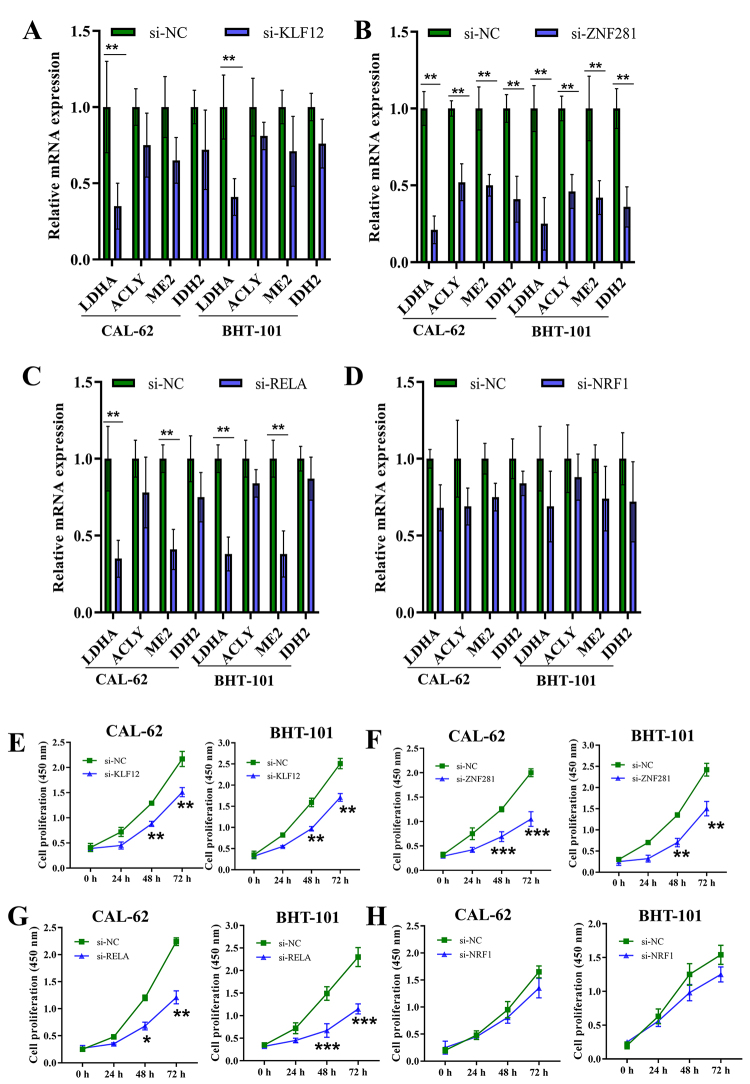
Effects of KLF12, ZNF281, RELA and NRF1 silencing on the expression of super-enhancer-controlled genes related to glucose metabolism and proliferation of PDTC cells. (A-D) Transcription of four representative genes (LDHA, ACLY, ME2 and IDH2) was detected using qRT-PCR after KLF12 (A), ZNF281 (B), RELA (C) and NRF1 (D) silencing. (E-H) Effects of KLF12 (E), ZNF281 (F), RELA (G) and NRF1 (H) silencing on cell proliferation was assessed using CCK8. ANOVA followed by Tukey’s post hoc test and Student’s *t*-test were used for statistical analysis. *P<0.05, **P<0.01, ***P<0.001.

Furthermore, we examined the transcription of four representative glucose metabolism-related genes controlled by super-enhancer (LDHA, ACLY, ME2 and IDH2) after silence of transcription factors. Silencing of KLF12 significantly downregulated LDHA expression (Figure 8A). Silencing of ZNF281 significantly inhibited LDHA, ACLY, ME2 and IDH2 expression (Figure 8B). LDHA and ME2 expression was significantly downregulated by knockdown of RELA (Figure 8C). However, silencing of NRF1 had non-significant effect on LDHA, ACLY, ME2 or IDH2 transcription (Figure 8D). Additionally, it can be seen that proliferation of CAL-62 and BHT-101 cells was significantly inhibited by silencing KLF12, ZNF281 or RELA, while there was no significant change in cell proliferation after silencing NRF1 (Figure 8E-H).

### Effects of KLF12, ZNF281 and RELA silencing on glucose metabolism in CAL-62 and BHT-101 cells

**Figure 9.**
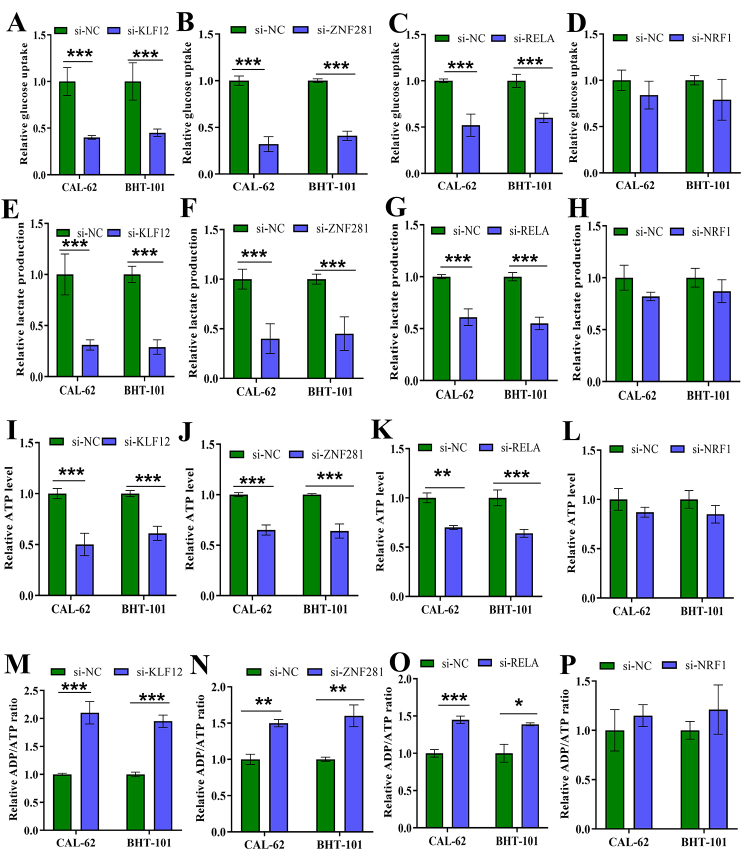
Effects of KLF12, ZNF281, RELA and NRF1 silencing on glucose metabolism in CAL-62 and BHT-101 cells. Relative glucose uptake (A-D), lactate production (E-H), ATP level (I-L) and ADP/ATP ratio (M-P) of CAL-62 and BHT-101 cells with or without KLF12, ZNF281, RELA and NRF1 silencing were measured. Comparison between two groups was conducted by Student’s *t*-test. *P<0.05, **P<0.01, ***P<0.001.

To more deeply explore the potential involvement of KLF12, ZNF281 and RELA in glucose metabolism, we examined the changes in glucose uptake in CAL-62 and BHT-101 cells after KLF12, ZNF281 and RELA silencing. Meanwhile, we selected NRF1, a transcription factor without significant effect on prognosis, as a control. Compared with si-NC group, transfection of si-KLF12, si-ZNF281 or si-RELA significantly suppressed glucose uptake capacity, while transfection of si-NRF1 had no significant impact on glucose uptake (Figure 9A-D). Glucose uptake by cells is followed by catabolism, which is the main way organisms obtain energy. Lactate is an important intermediate product of glucose metabolism. The investigation depicted that silencing of KLF12, ZNF281 and RELA significantly decreased the relative lactate production in both CAL-62 and BHT-101 cells (Figure 9E-G). However, silencing of NRF1 had no significant effect on lactate production (Figure 9H).

Furthermore, the relative ATP levels in si-KLF12, si-ZNF281 and si-RELA groups were notably lower compared to the control groups, while the reduction in ATP level in si-NRF1 group was not significant (Figure 9I-L). The relative ADP/ATP ratio were significantly increased after KLF12, ZNF281 and RELA silencing, whereas the increase in ADP/ATP ratio was not significant after silencing of NRF1 (Figure 9M-P). These data implied that silencing of KLF12, ZNF281 and RELA impaired cellular glucose metabolism in CAL-62 and BHT-101 cells.

## Discussion

Abnormal gene expression and metabolism profiles are major hallmarks of cancer. In the current study, transcription factors of glucose metabolism-related super-enhancer-controlled genes with prognostic value were filtered. Effects of these transcription factors on PDTC cells proliferation, glucose uptake, lactate production, ATP and ADP levels were verified *in vitro*.

Glucose metabolism plays a central role in overall cellular metabolism, and provides precursors and intermediates for other metabolism, as well as supplying energy to cell (Abdel-Wahab *et al.*, 2019). However, the molecular mechanisms of glucose metabolism reprogramming are largely unknown for PDTC. In this study, we performed an enrichment analysis of glucose metabolism-related pathways using GSEA, and found that the enrichment in TCA cycle and pyruvate metabolism was significant. Relationships between glucose metabolism and thyroid carcinoma differentiation are complex (Heydarzadeh *et al.*, 2020). In this study, we found that TCA cycle score and pyruvate metabolism score were significantly higher in PDTC than the other three (FTC, FVPTC and PTC) differentiation types.

TCA cycle supplies tumor cells with material and energy to facilitate metastasis (LeBleu *et al.*, 2014; Corbet and Feron, 2017; Cai *et al.*, 2020). In this study, the DEGs were located in many important reactions in TCA cycle. For example, malate dehydrogenase 2 (MDH2), ATP citrate lyase (ACLY) and citrate synthase (CS) were identified as DEGs, which are directly related to oxaloacetate metabolism. Oxaloacetate acts as an intermediate in many metabolism pathways such as glyoxylate metabolism, alanine aspartate and glutamate metabolism. Additionally, oxaloacetate also plays important roles in detoxification of reaction oxygen species (Sawa *et al.*, 2017). Fifteen DEGs were located in the pyruvate metabolism in this study. These DEGs were involved in the production of phosphoenol-pyruvate, oxaloacetate, acetyl-CoA, malonyl-CoA and others. These intermediates are in turning involved in many metabolisms. Lactate dehydrogenase A (LDHA) catalyzes the conversion of pyruvate to lactate, a reaction that occurs not only in pyruvate metabolism but also as a key final step in glycolysis. Activation of LDHA facilitates cancer cell invasion, anti-inflammation and metastasis (Jin *et al.*, 2017). A recent study has shown that high expression of LDHA enhances the proliferation and metastasis of PTC cells (Hou *et al.*, 2021). Hence, it is not difficult to find that the enzymatic reaction products mediated by DEGs (such as lactate, oxaloacetate and malonyl-CoA, etc.) have crucial effects on cellular metabolism, especially TCA cycle and pyruvate metabolism.

Compared to typical enhancers, super-enhancers are enriched with transcription factor binding sites, and drive target genes expression more powerfully (Sengupta and George, 2017; Tang *et al.*, 2020). Super-enhancers are critical regulatory elements that maintain the characteristic signaling pathways and metabolism of tumor cells (Thandapani, 2019; Sengupta and George, 2017). Herein, we identified the super-enhancers of CAL-62 cells. A total of 23 super-enhancer-controlled genes related to glucose metabolism in PDTC were screened out. Transcription factors regulate transcription of target genes by binding to super-enhancers, recruiting RNA polymerase II or elongation factors. Identification of transcription factors binding to super-enhancers is necessary to investigate the molecular regulatory mechanisms. We identified KLF12, ZNF281 and RELA as key enhancer drivers. Four glucose metabolism-related target genes associated with poor prognosis, LDHA, ACLY, ME2 and IDH2, were screened. Further experiments confirmed that the regulatory network of KLF12, ZNF281 and RELA can control the expression of these four prognostic target genes. These results suggested that KLF12, ZNF281 and RELA may regulate glucose metabolism and PDTC progression by driving super-enhancer reprogramming of LDHA, ACLY, ME2 and IDH2.

Kruppel like factor 12 (KLF12) has potential oncogenic effects in a variety of cancers (Jia *et al.*, 2019; Hou and Li, 2020; Mao *et al.*, 2020). A recent study has pointed out that KLF12 acts as an oncogene, and results to a poor outcome in PTC (Xu *et al.*, 2021). Similarly, we demonstrated that KLF12 expression was higher in PDTC cells than normal thyroid epithelial cells. Zinc finger protein 281 (ZNF281) has been demonstrated to promote metastasis and invasion in lung cancer, pancreatic cancer and colorectal cancer (Qian *et al.*, 2017; Xue *et al.*, 2019; Xian *et al.*, 2020). The current study shows that KLF12 and ZNF281 bind to the promoter regions of target genes to regulate their transcription (Mak *et al.*, 2017; Ji *et al.*, 2020). Our results suggested that the regulation of target genes by KLF12 and ZNF281 was not limited to binding in the promoter region, but have the ability to drive super-enhancer activation. RELA, an important enhancer driver, is a subunit of NF-Kappa-B that acts as a proto-oncogene in many cancers including PTC (Pyo *et al.*, 2013; Brown *et al.*, 2014; Lu and Yarbrough, 2015). Similarly, our study confirmed that RELA can drive glucose metabolism-related gene enhancer activation and promote PDTC progression. In addition, effects on metabolism of KLF12, ZNF281 and RELA in PDTC are poorly understood. In this study, we found that KLF12, ZNF281 and RELA expression were enhanced in PDTC cells compared with the normal thyroid epithelial cells. Functionally, PDTC cell proliferation was inhibited by silencing of KLF12, ZNF281 and RELA. Furthermore, KLF12, ZNF281 and RELA silencing resulted in a dramatic decrease in glucose uptake, lactate production and ATP level, but an increase in the ADP/ATP ratio of PDTC cells. More influences and detailed regulatory mechanisms of KLF12, ZNF281 and RELA on glucose metabolism in PDTC need to be further study.

## Conclusion

KLF12, ZNF281 and RELA were identified as the prognostic transcription factors of glucose metabolism related super-enhancer-controlled genes with potential as therapeutic targets for PDTC.
